# Vection Is Enhanced by Increased Exposure to Optic Flow

**DOI:** 10.1177/2041669518774069

**Published:** 2018-05-23

**Authors:** Takeharu Seno, Kayoko Murata, Yoshitaka Fujii, Hidetoshi Kanaya, Masaki Ogawa, Kousuke Tokunaga, Stephen Palmisano

**Affiliations:** Faculty of Design, Kyushu University, Minami-ku, Fukuoka, Japan; Graduate School of Humanities, Tokyo Metropolitan University, Hachioji-shi, Tokyo, Japan; Research Organization of Open Innovation and Collaboration, Ritsumeikan University, Ibaraki-shi, Osaka, Japan; Faculty of Design, Kyushu University, Minami-ku, Fukuoka, Japan; College of Comprehensive Psychology, Ritsumeikan University, Ibaraki-shi, Osaka, Japan; Faculty of Human Informatics, Aichi Shukutoku University, Nagakute-shi, Aichi, Japan; Faculty of Design, Kyushu University, Minami-ku, Fukuoka, Japan; School of Psychology, University of Wollongong, Wollongong, NSW, Australia

**Keywords:** vection, illusory self-motion, subjective strength, duration, latency, magnitude, animation

## Abstract

We examined whether vection strength could be modulated by altering the exposure duration to optic flow. Experiment 1 sourced 150 different video clips from various Japanese animation works which simulated self-motion. Despite large differences in the content of these video clips, we found a significant positive correlation between their play durations and their ratings of vection magnitude. Experiment 2 examined this relationship further using more tightly controlled visual motion stimuli. Vection was induced by presenting the motion of the same expanding grating stimulus for 8, 16, 32, or 64 seconds. While vection onset latencies remained constant across these four conditions, vection magnitude/strength was found to increase systematically with the exposure duration. As predicted by a recent computational model of vection, we conclude that subjective vection strength does depend on the exposure duration to optic flow.

## Introduction

When a large region of the visual field is stimulated by coherent motion, stationary observers often (illusorily and incorrectly) perceive that they themselves are moving (typically in the opposite direction to the stimulus motion). This type of visually induced illusion of self-motion has traditionally been referred to as *vection* (e.g., Brandt, Dichgans, & Koenig, 1973; Seno et al., 2017; for alternative uses of this term, see Palmisano, Allison, Schira, & Barry, 2015). Initially, observers tend to perceive this visual motion stimulation as being due to object or scene movement. Only later on will they begin to perceive this optic flow as being due to their own self-motion (Brandt et al., 1973; Warren, 1995). Despite this initial delay in induction, compelling vection can be subjectively indistinguishable from real self-motion (see Brandt, Wist, & Dichgans, 1971; Palmisano & Gillam, 1998). However, vection experiences are known to vary significantly and appear to depend on a variety of low-, mid- and even high-level factors in the visual motion stimulus.

Investigations of vection have traditionally focused on the low-level physical characteristics of the visual inducing stimulus (see Dichgans & Brandt, 1978 for a comprehensive review of this early research). For example, studies have shown that vection tends to increase with the size, density and speed of the visual motion stimulation. However, mid-level visual processing (see Anderson & Kim, 2009) also plays a role in vection. One recent study by Kim, Khuu, and Palmisano (2016) suggests that vection also depends on the computations involved in perceiving surfaces and material properties (i.e., it is not simply based on the low-level processing of retinal motion velocities). It is now also known that the experience of vection can even be influenced by higher level cognitive factors (for a review, see Riecke, 2010). For example, when the physical stimulus factors are held constant, naturalistic patterns of globally consistent motion stimulation often induce more compelling vection than spatially scrambled versions of these stimuli (e.g., Riecke, Schulte-Pelkum, Avraamides, Von Der Heyde, & Bülthoff, 2006).

What constitutes more compelling vection? In this past, it has been widely accepted that more compelling vection experiences are ones which generate stronger subjective ratings of vection magnitude, have shorter vection onset latencies and result in longer total durations of vection.^1^ However, the last of these three vection indicators assumes that the durations of the visual motion stimulation are the same for all of the experimental conditions being tested. This has led to one potentially important factor for vection (the exposure duration to the visual motion stimulation) being largely ignored. In past studies, this exposure duration was typically held constant (i.e., it was regarded as a fixed-factor) in order to allow other (low-, mid- or high-level) aspects of the visual motion stimulation to be systematically examined.

**Figure 1.**
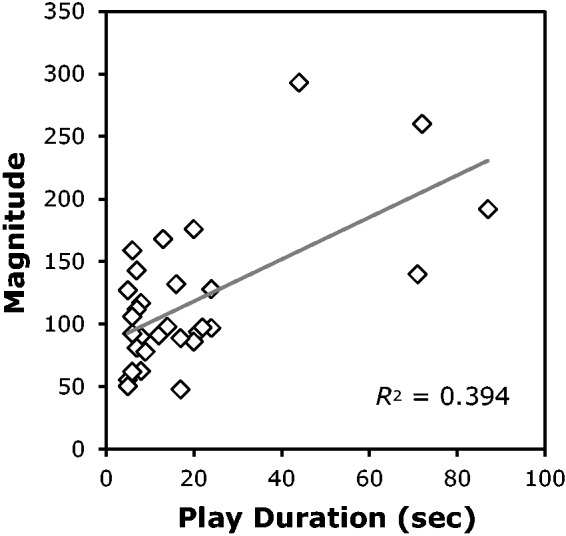
The relationship between the play durations of the different video clips and their rated vection magnitudes. This shows the data from 30 video clips obtained from Japanese animation films. The grey line indicates the linear regression line for this data. (We recreated this figure, based on the data set in Tokunaga et al. (2016), the original Figure number 3, on page 39.).

This study was interested in the effect that exposure duration might have on the experience of vection. A recent mathematical model of vection (the Oscillating Potential Vection Model [OPVM]; Seno et al., 2017) predicts that vection responses should increase with the duration of exposure to the optic flow. This prediction also appears to be supported by the findings of a recent study by Tokunaga, Ogawa, Ikehata, Masuda, and Seno (2016). This experiment examined the vection induced by 30 different video clips sourced from a variety of Japanese animation films (such as the Dragon Ball movies [Toei animation] and the Studio-GHIBLI-works directed by Hayao Miyazaki). While these film clips all provided coherent patterns of optic flow, they varied greatly in terms of both their actual content (e.g., flow types, speeds, densities, luminance, simulated material properties and surface optics, presence/absence of characters and their actions) and their playing times (the shortest of these clips had a play time of 4 seconds, whereas the longest had a play time of 87 seconds; hereinafter the playing time of these vection video clip is exclusively referred to as *play duration*). Despite presumably large differences in terms of the high- and low-level factors known to affect vection, the longer duration video clips were found to induce vection with significantly stronger magnitude ratings, *r* = .63, *t*(28) = 4.27, *p* < .001. Figure 1 shows this relationship.

The findings of Tokunaga et al. (2016) suggest that increasing the exposure duration to visual motion stimulation strengthens vection, irrespective of the nature and the content of the optic flow. However, it is premature to conclude that exposure duration plays a role in vection based on this data. As shown in Figure 1, most of the clips in this study had play durations which were less than 25 seconds. Therefore, some of these video clips might have been too short to reliably induce vection. Furthermore, only four clips had play durations longer than 40 seconds. Since these animation video clips were typically rather complicated visual motion stimuli (containing various uncontrolled [low-, mid- and high-level] stimulus parameters known to affect vection), it is possible that other extraneous stimulus factors (such as their flow speeds) were responsible for the stronger vection ratings found for these four longest play duration stimuli. Consistent with this alternative explanation, the positive correlation between vection magnitude and play duration became non-significant when the data for the four longest clips were excluded from the analysis, *r* = .17, *t*(24) = 0.82, *p* = 0.42. It should be noted that increasing the exposure duration to optic flow might also increase in the likelihood of vection dropouts (another complicating factor).

This study was aimed at testing the hypothesis that vection increases with exposure duration to the visual motion stimulation in two experiments.

Experiment 1 attempted to replicate and extend the findings of Tokunaga et al. (2016) by examining the strength of the vection induced by a much larger and more diverse selection of Japanese animation film clips. This new experiment tested the vection induced by 150 (as opposed to only 30) different video clips depicting coherent optic flow. As in the Tokunaga study, these stimuli were all sourced from contemporary Japanese animation films. However, this new stimulus set had a much wider range in terms of both stimulus content and play durations (minimum duration 4 seconds; maximum duration 106 seconds; average duration 19.1 seconds) compared to the stimuli in the earlier study. It was expected that the selection of such a large number of video clips would reduce the influence on vection of the other potentially confounding factors. It would also strongly demonstrate the generalisability of any motion exposure duration effect that was found for vection.

Experiment 2 took a different approach. This experiment examined the effect of motion exposure duration using a single, simple (i.e., clearly defined) vection inducing stimulus (presented for 8, 16, 32, or 64 seconds). Experiment 2 was designed to rule out the possibility that previous findings might have been due to artefacts present in the more complicated animation film clip stimuli used in Tokunaga et al. (2016) and Experiment 1. Furthermore, while these earlier experiments only obtained vection magnitude ratings (subjective strength of vection), Experiment 2 also examined the time course of this vection. The inclusion of a vection onset latency measure allowed us to rule out the possibility that our shorter video clips would not be able to reliably induce vection. Similarly, the inclusion of a vection duration measure allowed us to determine whether increasing exposure durations also increased the likelihood (and influence) of vection dropouts.

## Experiment 1

In this experiment, we examined the relationship between the exposure duration of the motion stimulation (play duration) and subjective vection strength (magnitude), using 150 different video clips with a wide range of play durations – all sourced from contemporary Japanese animation films (see supplementary material). The subjective magnitude of the vection induced by each of these different video clips was measured for each participant. We then calculated the correlation between the average play durations and the average vection magnitudes for these clips in order to compare our results with those of Tokunaga et al. (2016).

### Method

#### Ethics statements

The experiments in this article were pre-approved by the Ethics Committee of Kyushu University and were conducted following the guidelines of the Declaration of Helsinki. Written informed consent was obtained from each participant prior to the beginning of the experiment.

#### Participants

Twenty-two healthy adults including three authors (students, teachers and clerical staff of Kyushu University; 9 women and 13 men; the mean age was 24.05 years) participated in this experiment. All participants had normal or corrected-to-normal vision and no history of vestibular system diseases. The purpose of the experiment was not disclosed for them prior to the experiment.

#### Apparatus

Stimuli were controlled with a laptop computer (ALIENWARE M18x, Dell Inc., Round Rock, TX) and presented on a plasma display (3D VIERA TH-65AX800, Panasonic Corporation, Osaka, Japan; it had a 65 in. (165 cm) screen, a resolution of 1,920 × 1,080 pixels and a refresh rate of 60 Hz). The experiment was conducted in a dark room and participants sat on a rocking chair in order to enhance the induction of vection. Viewing distance was 57 cm. To promote vection, neither a chin-rest nor a head-rest was used.

#### Stimulus

One hundred fifty animation video clips from our new Vection Clip Database^2^ were used as the vection stimuli. The size of the motion field did vary across the clips, but a large (90%) area of the screen was always occupied by the motion in each case. Play durations ranged from a minimum of 4 seconds to a maximum of 106 seconds. The average play duration was 19.1 seconds (*SD* = 17.0). The resolution of each video clip was 1,024 × 768 pixels and the frame rate was 30 Hz. The different video clips were randomly presented by using the link transfer function in Microsoft Excel for Windows 2013. Each of the video clips was presented using a media player (Microsoft, ZuneVideo 2.6.344.0, x64).

**Figure 2.**
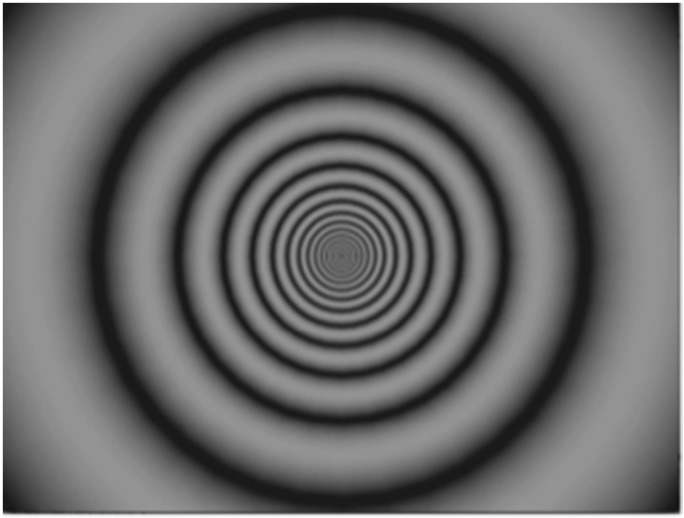
A frame of the standard stimulus used in Experiment 1. Participants observed this standard stimulus movie for 30 seconds.

To compare the subjective strength of the vection induced by these different video clips, an expanding circular grating was also used as a standard stimulus (see Figure 2). Stimulus spatial frequency decreased as eccentricity increased in order to induce a perception of forward self-motion. The maximum and minimum luminance values were 18.1 and 0.00 cd/m^2^, and Michelson contrast of the grating was 100%. The average grating speed was 25 deg/sec. Exposure duration was fixed at 30 seconds. Previous research has shown that this grating stimulus reliably induces moderately strong vection (e.g., Fukuda & Seno, 2012; Seno, Ito, Sunaga, & Palmisano, 2012).

#### Procedure

Participants binocularly observed the vection movie clips in an otherwise dark room. First, they observed the standard stimulus for 30 seconds, which was sufficient to establish the subjective strength of the vection it induced. If participants experienced vection during exposure to this standard stimulus, they were told that the subjective vection strength of this experience should be rated as ‘100’ (with ‘0’ representing no perception of self-motion). Subsequently, they estimated the subjective vection strength (magnitude) induced by each video clip by comparing this 100. For example, if the magnitude for a particular video clip was estimated to be twice as strong as that induced by the standard stimulus, the participant reported its magnitude as ‘200’. All 150 video clips were presented in a fully random order and estimated just once for each participant. They were presented as 15 blocks of 10 trials (with each trial presenting a different vection video clip). Participants were able to rest between trials. At the beginning of each block, the standard stimulus was shown again to prevent response drift in their magnitude ratings.

### Results and Discussion

Our participants’ average vection magnitude ratings ranged from 18.5 to 293. Vection was not reported in 8.4% trials. While these non-vection trials tended to have shorter play durations, none of the individual video clips tested obtained an average vection rating of less than 15 (indicating that at least some vection was induced by all of these video clips).

**Figure 3.**
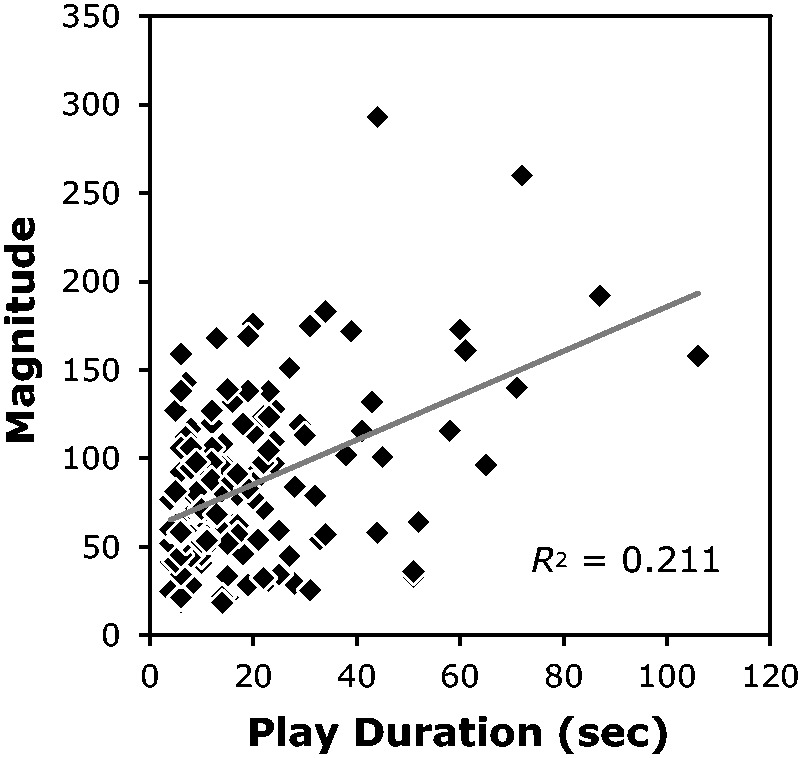
The correlation between play duration and vection magnitude obtained in Experiment 1. The grey line in the graph indicates the linear regression line for the data.

The average vection magnitude rating across all 150 vection clips was found to be 84.21 (*SD* = 46.50). Figure 3 shows the correlation between the play durations of these video clips and their rated vection magnitudes. The vection magnitudes that participants reported were found to be significantly and positively related to the play durations of video clips, *r* = .46, *t*(148) = 6.29, *p* < .001. In other words, as play durations increased, the ratings of vection magnitude tended to become larger. Tokunaga et al. (2016) reported the same tendency when they examined the vection induced by 30 video clips, but it was argued that this finding might have been due to pseudo-correlation (because of paucity of their data). However, our results, obtained from 150 video clips with a wider range of play durations, clearly replicate and confirm this previously observed positive correlation.

However, as noted in Introduction section, the animation video clips used in our experiment contained many components that could potentially influence the vection that our participants experienced, for example, motion signals, flow size and velocity, luminance, and characters. Therefore, it was still possible that these factors might have been confounded with the play durations (although the influence of these factors on the tendency of vection strength should have been reduced by analysing the data from 150 [as opposed to only 30] video clips). This relationship between play duration and magnitude needs clarification. Thus, in the next experiment, we further examined the relationship between exposure duration and vection using (a) better controlled vection stimuli and (b) additional vection indices.

## Experiment 2

The effect of the exposure duration of motion stimulus presentation (called *exposure duration*) on vection strength was investigated with simplified and tightly controlled motion stimuli. These vection stimuli were expanding circular gratings which were identical except for their exposure durations. Vection was induced on each trial by exposing participants to the motion of an expanding grating for 8, 16, 32, or 64 seconds. Like Experiment 1, vection magnitude ratings were obtained for each of these exposure duration conditions. However, this experiment also obtained measures of vection onset latency and vection duration as well. The former vection onset latency data allowed us to check that vection was still being induced in the shortest duration motion exposure trials. As the exposure duration of the visual motion stimulation varied from trial-to-trial, the vection duration for each trial in this experiment was calculated instead as a percentage:

$$\%-\mathrm{duration}=\frac{\mathrm{Total}\;\mathrm{accumulated}\;\mathrm{duration}\;\mathrm{pereceiving}\;\mathrm{vection}}{\mathrm{Motion}\;\mathrm{exposure}\;\mathrm{duration}}$$

This %-duration measure has been used previously by Keshavarz, Speck, Haycock, and Berti (2017). Not only does this %-duration measure provide an alternative index of the strength of vection, it can also be used to provide information about the contribution of vection dropouts to exposure duration effects.

### Method

#### Participants

Nineteen healthy adults (students of Kyushu University; 3 women and 16 men; the mean age was 25.36 years) participated in this experiment. Three of these individuals had previously participated in Experiment 1. All participants had normal or corrected-to-normal vision and no history of vestibular system diseases. They were also naïve as to the purpose of the experiment.

#### Apparatus

The equipment was the same as Experiment 1, with the following exception: Stimuli were controlled via a desktop computer (Precision T3610, Dell Inc., Round Rock, TX).

#### Stimulus

**Figure 4.**
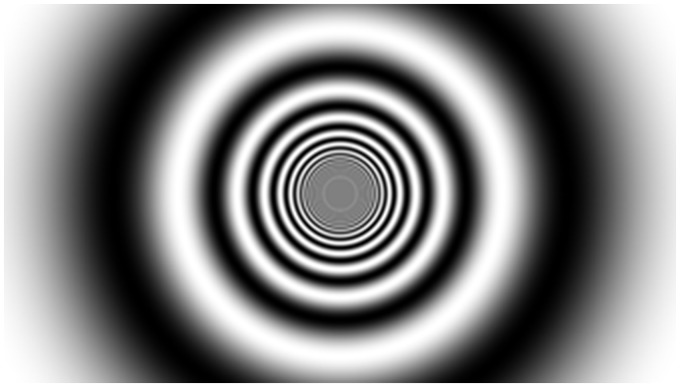
A frame of one of the vection stimuli used in Experiment 2. These displays were generated by code written for MATLAB 7.11.0.584 (R2010b) and which also utilised PsychToolbox-3 (Brainard, 1997; Kleiner, Brainard, & Pelli, 2007; Pelli, 1997).

The standard stimulus display in this experiment was the same as that used in Experiment 1. The vection stimuli were identical to those previously used in Fujii, Seno, and Allison (2018) – except that four different motion exposure duration conditions were examined (8, 16, 32, and 64 seconds). They were all expanding grating movies, which were defined by luminance modulation (Figure 4). The maximum and minimum luminance values in these displays were 16.2 cd/m^2^ and less than 0.01 cd/m^2^, respectively; the Michelson contrast was 100%. Each of these movies simulated forwards self-motion through a 3D cylinder covered with a sinusoidal grating texture (its simulated radius was 266 cm and the wavelength of the texture was 444 cm). The speed of simulated self-motion in depth was approximately 444 cm/sec. A grey disc was placed in the centre of the screen to occlude spatial aliasing artefacts. This disc was static in the centre of the display (no other fixation target was presented). These motion stimuli subtended a visual area 100 deg wide by 81 deg high at the viewing distance of 57 cm.

#### Procedure

Participants sat on a chair in a dark room and observed the motion stimuli. They were instructed to gaze binocularly at the centre of stimuli whenever a motion stimulus was presented. They were also instructed to press and hold a response button whenever they perceived self-motion (i.e., vection) in order to measure vection onset latency and the duration of vection. After each trial, a rating of vection magnitude was also obtained. Participants rated subjective vection magnitude using a 0 to 100 rating scale (where 0 represented *no vection* and 100 represented *very strong vection*). We should clarify here that we used a different magnitude rating method from that of Experiment 1 (in this experiment, no standard stimulus was used). Very strong vection meant that participants perceived self-motion very naturally, as if they were moving throughout the stimulus presentation. Each participant was presented with all four exposure duration conditions in random order, and repeated each of these conditions four times. Thus, 16 trials were conducted in total (per participant).

### Results and Discussion

**Figure 5.**
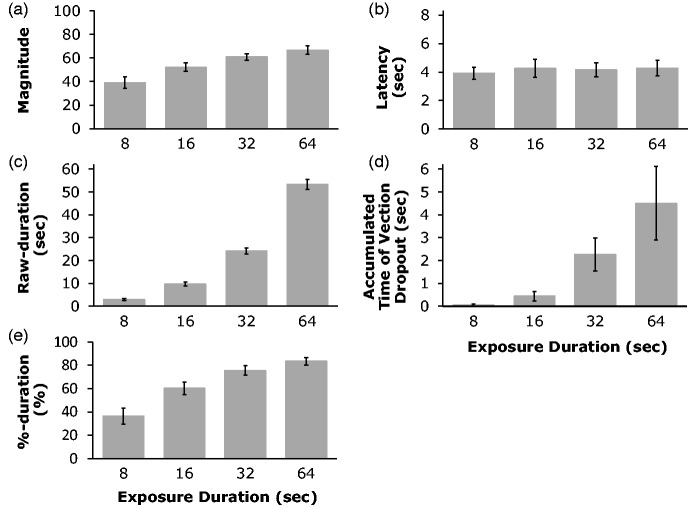
The results of Experiment 2. (a) Vection magnitudes, (b) Vection onset latency, (c) Raw-duration, (d) Vection dropout, and (e) %-duration. Error bars show 1 *SE*s.

Figure 5 shows the results of Experiment 2. Figure 5(a) shows that vection magnitude increased significantly with exposure duration to the motion stimulation, *F*(3, 54) = 22.20, *p* < .001. Ryan’s method revealed that there were significant differences between all combinations of the four conditions except between the 32- and the 64-second conditions (*ps* < .05). The later null finding for the 32- and the 64-second conditions suggests that vection strength might have been either saturated or close to saturation for the 32-second condition. This saturation in vection strength has been previously reported by other studies (e.g., Apthorp & Palmisano, 2014).

In the shortest (8-second) exposure duration condition, vection was reported in 71% (i.e., 54/76) of trials and the average vection onset latency was approximately 4 seconds (Figure (5b)). These results indicated that substantial vection was induced even with this rather short exposure duration. Vection was reported in 97.4% (i.e., 74/76) of trials for the 16-second condition and in 100% (76/76) of the trials for both 32- and 64-second conditions. When non-vection trials occurred they appeared to be due to individual differences in vection onset latency. Four of our participants did not report any vection during any of their 8-second condition trials; their averaged onset latency for the longer exposure duration conditions was 10.2 seconds. This suggests that it took longer than 8 seconds for these participants to experience vection (i.e., the stimulus stopped moving before they started to experience vection in the 8-second condition). With the exception of these four individuals, our results show that vection could still be induced by the shorter exposure durations. This in turn suggests that the shorter play durations in the video clips used in Experiment 1 could still have induced substantial vection.

As noted above, onset latency data were not obtained for four participants in the 8-second condition. The latency data were also lost for one additional participant due to recording problems. Therefore, the latency data in the 8-second condition for five participants were excluded from the following analyses described below, that is, Figure 5(b), the averaged onset latency for four exposure duration conditions. We found that there was no significant difference in the vection onset latencies for four different exposure duration conditions, *F*(3, 39) = 0.42, *p* = .74. The stimuli used in these four conditions were the same except for their exposure durations. Therefore, the influence of low-, mid- and high-level stimulus factors on vection induction should have been initially the same for all four conditions. Thus, it was expected that there should be no difference in the vection onset latencies in the four conditions (as the benefits of the progressively longer exposure durations would only emerge later on in the trial).

**Figure 6.**
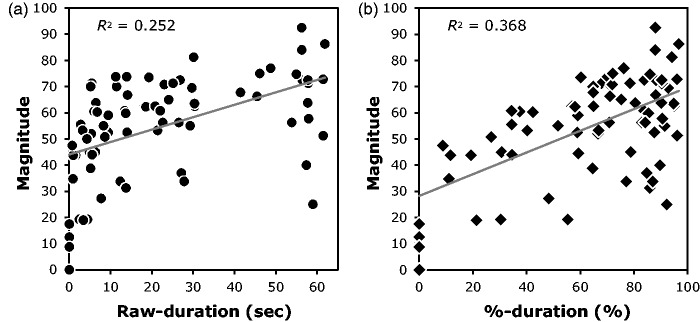
The relationships between (a) the Raw-duration and magnitude-based vection measures and (b) the %-duration and magnitude-based vection measures. The grey line in each graph indicates the linear regression line for each data.

We next looked at the Raw-duration vection measure (i.e., the total [accumulated] amount of time that vection was experienced during the stimulus presentation). As expected, Figure 5(c) shows that this Raw-duration vection measure increased significantly with the exposure duration, *F*(3, 54) = 633.22, *p* < .001. Ryan’s method revealed that there were significant differences between all combinations of the four conditions (*ps* < .001). Because this tendency was similar to that found for the rated vection magnitude, we conducted a correlational analysis between the Raw-duration of vection and the rated vection magnitude. Figure 6(a) shows a significant positive correlation between these two vection measures, *r* = .50, *t*(74) = 4.99, *p* < .001.

It was also possible for vection to drop out during stimulus presentation – such dropouts could occur after the onset of vection (the first button press) and before the end of the motion stimulus presentation. For example, if the induced vection was very weak or ambiguous. In this experiment, vection dropouts occurred in 1, 9, 23 and 33 of 76 trials in 8-, 16-, 32- and 64-second conditions, respectively. Figure 5(d) shows the averaged accumulated time of the vection dropouts for each of the exposure duration conditions. These results show that as the exposure duration increased, vection dropouts tended to last longer and become more frequent. A one-way, repeated-measures ANOVA on accumulated vection dropout times revealed a significant main effect of the exposure duration, *F*(3, 54) = 6.85, *p* < .001. This pattern of results had been previously assumed by our computational model of vection responding (see OPVM; Seno et al., 2017). The relationship between the predictions of OPVM and the present data is mentioned in the General Discussion section.

As both Raw-duration and vection dropouts both increased with the exposure duration, we also examined these exposure duration effects on vection using another index of vection strength (*%-duration*). %-duration indicates the percentage of time that vection was experienced as a function of the exposure duration for the particular trial. Figure 5(e) shows that this %-duration vection measure also increased with the exposure duration. The main effect of exposure duration was significant, *F*(3, 54) = 62.41, *p* < .001. Ryan’s method revealed that except for the difference between the 32- and the 64-second conditions, all other combinations of the four exposure duration conditions were significant (*ps* < .001). The effects of exposure duration on vection magnitude ratings and this %-duration vection measure were also very similar. Thus we also conducted a correlational analysis between these two vection measures (Figure 6(b)). The results showed that these two measures were significantly and positively correlated, *r* = .61, *t*(74) = 6.55, *p* < .001. Although the R value for this correlation was larger than that for the correlation between Raw-duration and magnitude, these two R values were not found to be significantly different, *t*(74) = 1.44, *p* = .15. This implies that %-duration and Raw-duration had nearly the same validity as vection measures in the current experiment.

## General Discussion

In this study, we examined the relationship between the exposure duration to visual motion stimulation and the strength of the induced vection. Experiment 1 followed a similar procedure to Tokunaga et al. (2016). We showed participants 150 different vection video clips (all sourced from Japanese animation films) and measured the subjective vection magnitude ratings for these clips. Overall, our results indicated that clips with longer play durations generated stronger subjective vection magnitude ratings (despite the other differences in terms of the content of these clips). These exposure duration effects on vection were replicated in Experiment 2 using more tightly controlled vection stimuli (the same expanding grating stimulus was presented on all trials but with different exposure durations). We found that the subjective magnitude of the vection induced by this visual motion display increased with the reported duration of vection (vection magnitude ratings correlated almost equally with both Raw-duration and %-duration vection time course measures). Thus, the main finding of these two experiments was that vection strength can be still enhanced (considerably after the initial onset of vection) by increasing the exposure duration to the motion stimulation.

We recently outlined a mathematical model of vection responding (OPMV; see Seno et al., 2017) which assumed the following three temporal characteristics of vection: (a) there is a finite delay of several seconds between the start of the visual motion stimulation and observers’ first report of vection experience, (b) vection strength increases over time after the onset of vection, and then the strength eventually plateaus, and (c) vection dropouts can often occur (e.g., Dichgans & Brandt, 1978; Howard, 1982; Riecke, 2010). These assumptions were empirically confirmed by manipulating the exposure (and play) durations in the present study. In particular, we found that the number and the total periods of vection dropouts increased as the exposure (and play) durations became longer. These dropouts have not received much attention in most previous vection studies. Thus, future research into vection dropouts might further clarify the temporal properties of vection experience.

Raw-duration and vection dropouts were both found to increase with the exposure duration to the motion stimulation. Thus, we also calculated %-duration as an alternative index of vection strength. %-duration was also found to correlate significantly and positively with ratings of vection magnitude. While %-duration and Raw-duration both appeared to be valid indices of vection strength, %-duration would appear to be the more useful index of vection in this type of experiment for the following reasons. First, %-duration can be used to more readily compare the vection strengths induced by stimuli with very different exposure durations. By using %-duration, researchers should also be able to compare the vection induced in different past studies (even if they use very different stimuli and had very different exposure durations). This particular benefit has the potential to significantly advance future vection research. Second, %-duration is determined by several temporal aspects of vection. Specifically, %-duration will decrease as the vection onset latency and the number of dropouts for the trial both increase, and also as the raw-duration for the trial decreases. Thus, %-duration should also provide a more integrated index of vection strength (than any of these more traditional/common measures on their own). The correlation R value between %-duration and magnitude was slightly higher than that between Raw-duration and magnitude, although the results of the comparison between these two R values did not show a clear statistical difference. Considering these two viewpoints, %-duration would be a very effective and the most valid vection index.

**Figure 7.**
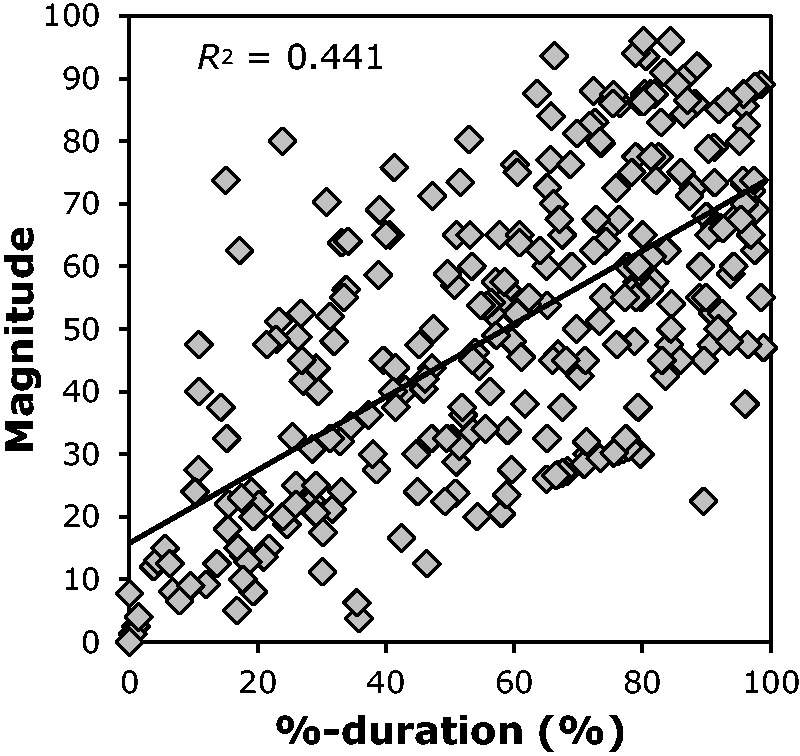
The correlation between %-duration and magnitude by using 317 individual data sets used in Seno et al. (2017). The black line indicates the linear regression line for the data.

Recently, Seno et al. (2017) analysed 317 data sets, each consisting of three vection indices (vection onset latency, vection duration and rated vection magnitude), obtained in different seven vection experiments (the exposure duration of the motion stimuli was fixed at 40 seconds in all of these experiments). Vection onset latencies in these experiments ranged from 0.46 and 40 seconds. Figure 7 shows that, in these experiments, %-duration correlated significantly with the rated magnitude of vection (*r* = .66, *t*(315) = 15.81, *p* < .001). From the results, it might be argued that these two indices (magnitude and %-duration) are redundant. However, it should be noted that %-duration was obtained via button pressing, whereas magnitude was obtained via subjective estimation. It has been argued that time course measures should be less affected by experimenter demands and cognitive biases than verbal/other ratings of vection strength/magnitude (e.g., Palmisano & Chan, 2004). Thus, although these two indices were highly correlated, they were obtained using totally different methodologies. In order to get a better estimate of the participant’s inner subjective experience, we believe that all possible methods/indices to assess vection should be used (to provide convergent evidence). Therefore, the high correlation between them instead suggests that these measures both appropriately reflect the participant’s inner vection strength.

The present results also provide insight into the basis of these subjective estimates of vection strength. The OPVM assumes that vection magnitude corresponds to the convolution of moment-to-moment subjective vection strength (Seno et al., 2017). This mathematical model predicts that when exposure duration increases, vection magnitude should also increase. However, if the reported vection magnitudes were instead estimated based either on the peak strength of the vection experience or on the vection inducing potential of the stimulus itself, then vection magnitudes should have remained constant irrespective of the exposure duration (particularly in Experiment 2 when exactly the same vection inducing stimulus was used for all conditions). Contrary to the latter hypothesis, the present results showed that the rated vection magnitude and %-duration were highly and positively correlated. These results were therefore consistent with the predictions of OPVM. They suggest that participants used %-duration when they estimated vection magnitude. In other words, they estimated vection magnitude by taking into account how long they perceived vection and how long the vection dropouts lasted.

There was another possibility about %-duration that participants did not consider the length of latency in the rating of vection magnitude. If this was the case, the ratio of vection duration to *the stimulus duration minus latency* should be important for evaluating vection magnitude. We named this the *Duration extracted %-duration*. However, when we conducted a correlation analysis between vection magnitude and this Duration extracted %-duration, no significant correlation was found between them (*r* = .17, *t*(69) = 1.44, *p* = .15). We conclude that the length of latency is included in estimates of vection magnitude. This finding further supports the notion that %-duration provides a superior index of vection magnitude.

In summary, %-duration appears to be a useful and valuable new index of vection. It is compatible, at least partially, with rated vection magnitude. The three vection indices used in previous vection studies tend to correlate with one another (i.e., rated vection magnitude, vection onset latency and Raw-duration; Seno et al., 2017) and this new index also correlates well with these three traditional measures. This new index %-duration used in Keshavarz et al. (2017) and in the present study could help reveal new aspects of vection. The validity and availability of this index should be further examined in future research.

We would like to conclude by discussing some of the possible applied applications of this research. Over the years, vection has played an important role in the entertainment industry and animation (anime in particular, as is evidenced by the 150 different vection-related anime video clips that we were able to amass for testing in Experiment 1). However, many of the important features of these movie/animation stimuli are absent in traditional laboratory experiments on vection, which are typically focussed on the vection induced by rather artificial dot motions. For example, animation typically contains an abundance of scene content with material properties that vary in terms of their surface optics (such as whether they are matte or glossy). The contributions that these extra scene details and information make to vection have received surprisingly little examination (with the notable exceptions of recent studies by Kim et al., 2016 and Ogawa, Hiramatsu, & Seno, 2014). While it is clearly more difficult to study vection (and perception in general) using realistic videos and cartoon animations, we have shown here that it is possible. Our extensive Vection Clip Database will hopefully provide researchers with interesting opportunities to explore the phenomenon of vection in the future as well as contribute to the world-wide effort for cultural heritage preservation.

## Supplemental Material

Supplemental material for Vection Is Enhanced by Increased Exposure to Optic Flow Click here for additional data file. Supplemental material, for Vection Is Enhanced by Increased Exposure to Optic Flow by Takeharu Seno, Kayoko Murata, Yoshitaka Fujii, Hidetoshi Kanaya, Masaki Ogawa, Kousuke Tokunaga and Stephen Palmisano in i-Perception

## Author’s Note

Kayoko Murata is also affiliated to Research and education center for natural sciences, Keio University, Yokohama-shi, Kanagawa, Japan. Masaki Ogawa is also affiliated to Faculty of Engineering, Mie University, Tsu-shi, Mie, Japan.

## Notes

1 The most commonly obtained measures of vection have been (a) the latency to vection onset (i.e., the delay between the start of the visual motion stimulation and the observer’s first reported experience of illusory self-motion), (b) the total duration of the vection (i.e., the total amount of time that the observer reported experiencing vection during the trial) and (c) magnitude estimates/ratings of the vection experience (e.g., verbal ratings using a linear scale from “0” = *no vection* to “100” = *very strong vection*), see Seno et al. (2017).

2 This information is also provided in the Supplementary Material. The 150 video clips used in Experiment 1 also included the 30 clips used in the earlier study by Tokunaga et al. (2016). All of the relevant information about these 150 vection video clips is provided in the following link (Microsoft Excel File Format): http://www.senotake.jp/dl/VectionDBENG23.xlsx
